# Perceptions of Regulatory Decision-Making for New Drugs From the Viewpoints of the Manufacturers in South Korea

**DOI:** 10.3389/fmed.2022.869262

**Published:** 2022-03-30

**Authors:** Kyung-Bok Son, Sylvia Park

**Affiliations:** ^1^College of Pharmacy, Hanyang University, Ansan, South Korea; ^2^Korea Institute for Health and Social Affairs, Sejong City, South Korea

**Keywords:** regulatory decision-making, manufacturer, perceptions, qualitative research, South Korea

## Abstract

Regulatory decisions for new drugs approval present high uncertainty, low reversibility, the avoidance of observable errors, and high political stakes. However, research on the behavior of regulatory agencies is scarce, particularly in the context of more open decision-making processes. We aimed to evaluate the perceptions of regulatory decision-making for new drugs approval from the viewpoints of the manufacturers in South Korea. In 2019, employees in domestic (*n* = 5) and foreign (*n* = 7) manufacturers with expertise in regulatory affairs were invited to participate in a questionnaire survey and semi-structured group interview. We asked about the relevance of various criteria in regulatory decision-making, the participation of various stakeholders, and the degree of consent for new drug approval with uncertainty. The domestic and foreign manufacturers perceived that a regulatory decision made by the MFDS was solely based on technical merit within a closed decision-making system. They responded that safety, efficacy, and benefit-to-harm ratio were the most relevant criteria and the most prioritized criteria in regulatory decision-making. They also perceived that the MFDS was the sole relevant member in a regulatory decision. However, the foreign manufacturers disagreed that the regulatory agency and the advisory committee were independent of conflicts of interest, which might imply that regulatory decisions were occasionally determined by the agency given the political benefits and/or costs within a more open system. The role of an advisory committee in terms of deliberation and participatory democracy were requested to make politically legitimate regulatory decisions from the viewpoints of the manufacturers. However, their perceptions toward public involvement in regulatory decision-making is still at the early stage.

## Introduction

The pharmaceutical sector is one of the most highly regulated markets (1). Regulatory decisions (or new drug reviews) are essential for new drugs to be distributed under health systems (2). Manufacturers are required to provide substantial evidence regarding the safety and efficacy of new drugs (3), and then regulatory agencies review the submitted evidence with in-house expert employees (4). Sometimes, regulatory agencies consult advisory committees to seek their expertise in rapidly changing knowledge and technology areas (5). The agency's decision is essentially final as well as immensely consequential (6, 7). Contesting a regulatory decision is difficult and time- and cost-consuming, and regulatory decisions consequently shape the internal and external market (8).

Regulatory decisions for new drugs approval present high uncertainty, low reversibility, the avoidance of observable errors, and high political stakes (6). These characteristics imply that an agency's decision-making might be politically shaped by the involvement of various stakeholders or interests groups (9). To address this issue, regulatory agencies can develop or refine their bureaucratic strategies. Previous research emphasized “reputation” to understand the behavior of regulatory agencies (10). Many researchers have argued that regulatory agencies have developed strategies to enhance their reputations and protect them from reputational threats (11–13). In contrast, there have been requests to create inclusive, transparent, and deliberative systems for decision-making (13–16). Stakeholders' involvement and their embedded roles have been a common practice in various health sectors.

### Regulatory Decision-Making for New Drugs in South Korea

Regulatory decision-making for drugs in South Korea had been under jurisdiction of the Ministry of Health. Meanwhile, Korea Food and Drug Administration (KFDA) was established in April 1996 to oversee food and drug safety, and it was promoted to the Ministry of Food and Drug Safety (MFDS) in March 2013 (17). In terms of approving new drugs, there has been a continuous concern on delayed marketing approval compared to other high-income countries (18) and lack of human resources and expertise in a decision-making body (19). In June 2021, the South Korean government prepared “the bio-health regulatory science development strategy” to enhance access to innovative new drugs and to secure competitiveness in national bio-health industry (19). At the center of the strategy, accelerating the marketing approval for innovative new drugs lies.

Regulatory decision-making process in the MFDS consists of three stages (20), which is very similar to that of other agencies (21, 22). First, a regulatory agency receives an application submitted by a manufacture and the agency evaluates the safety, efficacy, and quality of the data included in the application (20). Next, the regulatory agency can decide whether or not to refer the application to an advisory committee for a consultation. Finally, the regulatory agency evaluates the application with in-house expert reviewers or sometimes with the aid of an advisory committee. Regulatory agency experiences a challenge in maintaining in-house experts for reviewing the applications. It is difficult for the agency to hire additional employees. Furthermore, the regulatory agency cannot compete with the private sector to recruit capable reviewers (23). In these circumstances, the regulatory agency turns to an advisory committee to supplement its expertise.

This study analyzed the behavior of regulatory agencies (6), which have been requested for inclusive, transparent, and deliberate processes for regulatory decision-making (24). Research on regulatory agencies in the context of more open decision-making processes is scarce. Furthermore, manufacturers are major stakeholders in the regulatory decisions. However, their perceptions toward regulatory decision-making have not been comprehensively reported yet. We aimed to evaluate the perceptions of regulatory decision-making for new drugs from the viewpoints of the manufacturers in South Korea. This study could shed light on establishing politically legitimate regulatory decision-making processes for new drugs approval.

## Materials and Methods

### Study Design and Subjects

We conducted this study as part of a larger study on the perceptions of decision-making for adopting new drugs from the viewpoints of the manufacturers. Their perceptions toward reimbursement decision-making for new drugs approval have been published elsewhere (25). This study evaluated manufacturers' perceptions of regulatory decision-making.

We conducted a questionnaire survey and a semi-structured group interview designed for employees in manufacturers. The study subjects were employees in domestic and overseas manufacturers who had expertise in regulatory affairs. More specifically, they had at least 10 years of working experience on the related field and had extensive experience in introducing new drugs into the South Korean market. Note that the number of manufacturers, in particular domestic manufacturers, who had introduced new drugs into the market was limited. We contacted them through e-mails and asked for their participation in this study. If they could not participate, we asked them to recommend another relevant person in the organization. A total of 12 interviewees from five domestic and seven foreign manufacturers were recruited and interviewed from May 28, 2019, to June 27, 2019. This study was approved by the Institutional Review Board (IRB) of Ewha Womans University (IRB No. EWHA-201904-0010-01).

### Survey Questionnaire

The survey questionnaire was designed to evaluate decision-making for new drug approval from the viewpoints of manufacturers. The questionnaire was composed of four sections (Supplementary Table 1). First, we asked about various criteria in regulatory decision-making processes. We proposed 16 criteria and asked about their relevance and priority in regulatory decision-making. The criteria were categorized into the characteristics of drug, disease, and status in other countries. Second, we asked about the participation of various stakeholders in the decision-making process. Stakeholders were categorized into interest groups, expert groups, and government authorities. We asked about their participation in decision-making processes in terms of relevance, interests, and influences. Third, we created several scenarios regarding the characteristics of new drugs and asked the degree of consent for their market approval. The scenarios were presented in two ways, from the perspectives of uncertainty in safety and efficacy and the expected benefits and risks. The degree of consent for market approval in each scenario was measured as a binary variable (1 for market approval and 0 for non-market approval). Finally, we asked about decision structure, transparency, regulations, and stability of the regulatory decisions. A 5-point Likert scale from−2 (never relevant) to 2 (very relevant) was used to rate the survey items.

## Results

Table 1 presents the relevance of 16 criteria in regulatory decision-making. The domestic and foreign manufacturers indicated that safety, efficacy, and benefit-to-harm ratio were the most relevant criteria (rated more than 1.70 pts) in regulatory decision-making. Foreign manufacturers also indicated that the consistency of the evidence, disease severity, and marketing approval in other countries were relevant criteria (rated more than 1.00 pts). However, domestic manufacturers rated no other items as relevant criteria (rated more than 1.00 pts). We also asked about the 1st, 2nd, and 3rd prioritized criteria in regulatory decision-making, and assigned them 3 points, 2 points, and 1 point, respectively. The domestic manufacturers perceived efficacy (13 pts), safety (12 pts), and benefit-to-harm ratio (6 pts) as the most prioritized criteria, while the foreign manufactures perceived the benefit-to-harm ratio (13 pts), safety (11 pts), and efficacy (8 pts) as the most prioritized criteria.

**Table 1:** Relevance of criteria in regulatory decision-making on new drugs.

**Criteria**		**Domestic *N* = 5**	**Foreign *N* = 7**
Drug	Safety	1.80	1.86
	Efficacy in clinical trials	1.80	1.71
	Clinical effectiveness in real world	0.25	0.86
	Benefit-to-harm ratio	1.80	1.86
	Consistency of evidence	1.00	1.29
	Price/cost of treatment	−1.60	−1.00
	Cost effectiveness	−1.40	−1.00
	Budget impact	−1.40	−1.14
Disease	Disease severity	0.40	1.57
	Health-related quality of life	0.00	0.57
	Alternative treatment	−0.40	0.29
	Burden of disease	−0.20	0.43
	Patient population	0.20	1.00
Status in other	Marketing approval in other countries	0.80	1.14
countries	Reimbursement status in other countries	−1.40	−1.00
	Price in other countries	−1.40	−1.14

Table 2 presents the relevance of the participation of various stakeholders in a decision-making body and advisory board. A value more than 1 point was assumed as relevant. The manufacturers perceived that the MFDS was a relevant member in a decision-making body and the remaining stakeholders were not relevant members. The manufacturers responded that the members of the expert group, excluding experts in public health, were relevant members of an advisory body. Variations in the perceptions were also noted. Domestic manufacturers perceived an expert in public health as a relevant member of an advisory board, while foreign manufacturers perceived a patient as a relevant member of an advisory board. The manufacturers responded that laypersons were the most irrelevant members in a decision-making body.

**Table 2:** Relevance of the participation of various stakeholders in a decision-making body and advisory board.

		**Domestic** ***N*** **=** **5**		**Foreign** ***N*** **=** **7**	
		**Decision body**	**Advisory board**	**Decision body**	**Advisory board**
Interest groups	Manufacturers	0.20	0.20	−0.43	0.57
	Consumer groups	−1.40	−1.25	−0.57	0.29
	Patient groups	−0.25	0.25	0.00	1.29
	Laypersons	−1.60	−1.25	−1.57	−0.71
Expert groups	Physicians	0.80	1.20	0.71	1.86
	Toxicologist	0.60	1.60	0.71	1.14
	Clinical Pharmacy	0.60	1.60	0.57	1.43
	Statistics	1.00	1.60	0.43	1.43
	Public Health	0.20	1.20	0.00	0.86
Government authority	MFDS	1.90	1.20	2.00	0.57
	HIRA	−0.80	0.00	−1.00	−0.43
	NHIS	−0.80	−0.60	−1.29	−1.43
	MOH	−1.00	−0.40	−0.86	−0.14

*MFDS, Ministry of Food and Drug Safety; HIRA, Health Insurance Review and Assessment Service; NHIS, National Health Insurance Service; MOH, Ministry of Health and Welfare*.

**Figure 1:**
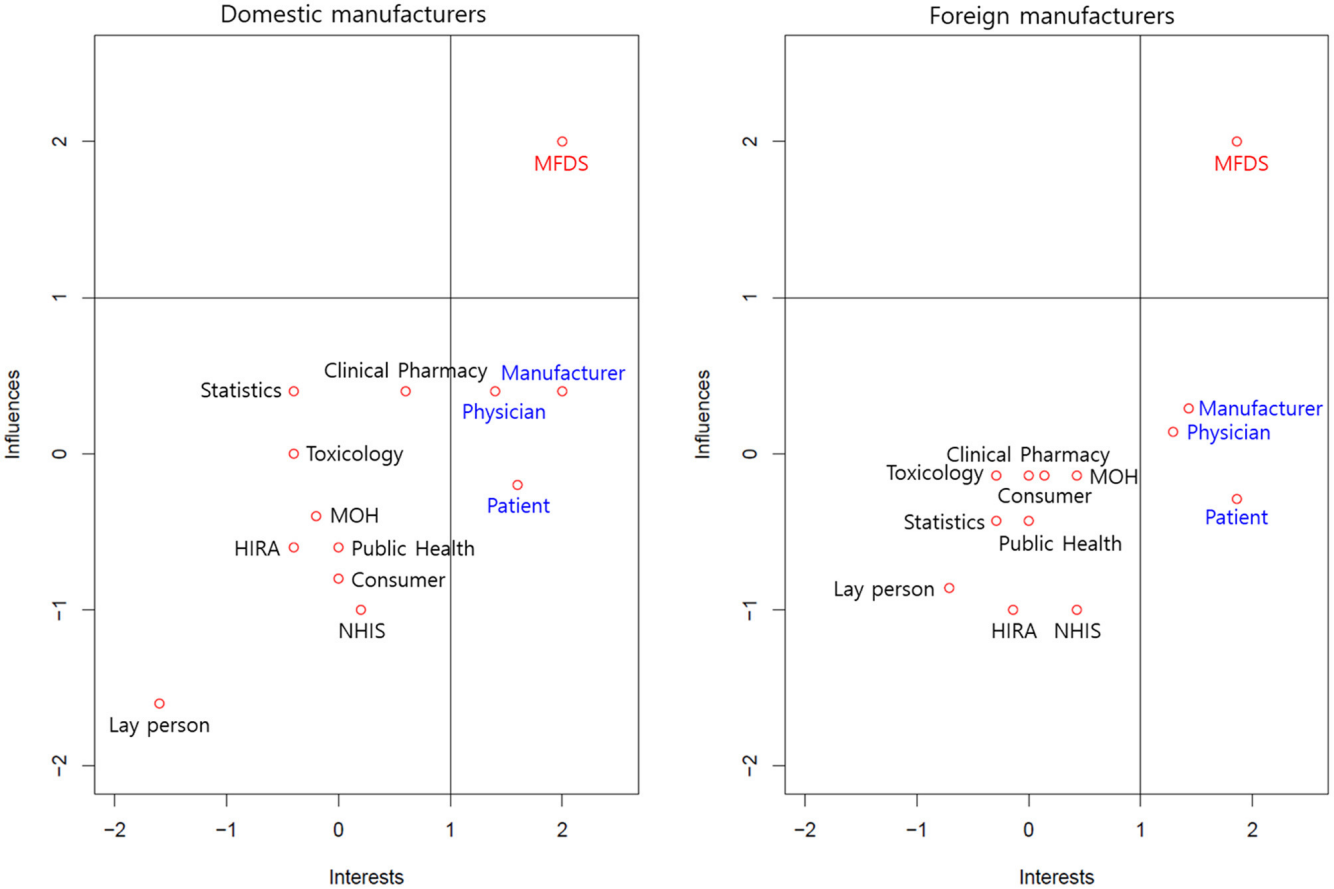
Interests and influences of various stakeholders in regulatory decisions. MFDS, Ministry of Food and Drug Safety; HIRA, Health Insurance Review and Assessment Service; NHIS, National Health Insurance Service; MOH, Ministry of Health and Welfare.

Figure 1 describes the perceived interests and influences of various stakeholders in regulatory decision-making. We defined a value rated more than 1 point as strong and categorized the 13 stakeholders into three groups: the group with strong interests and strong influences; the group with strong interests but weak influences; and the group with weak interests and weak influences. The manufacturers perceived the MFDS as a sole group with strong interests and strong influences. Similarly, they perceived manufacturers, physicians, and patients as a group with strong interests but weak influences. The remaining stakeholders were described as a group with weak interests and weak influences. Interestingly, the manufacturers perceived laypersons as a group with the weakest interests and influences.

**Figure 2:**
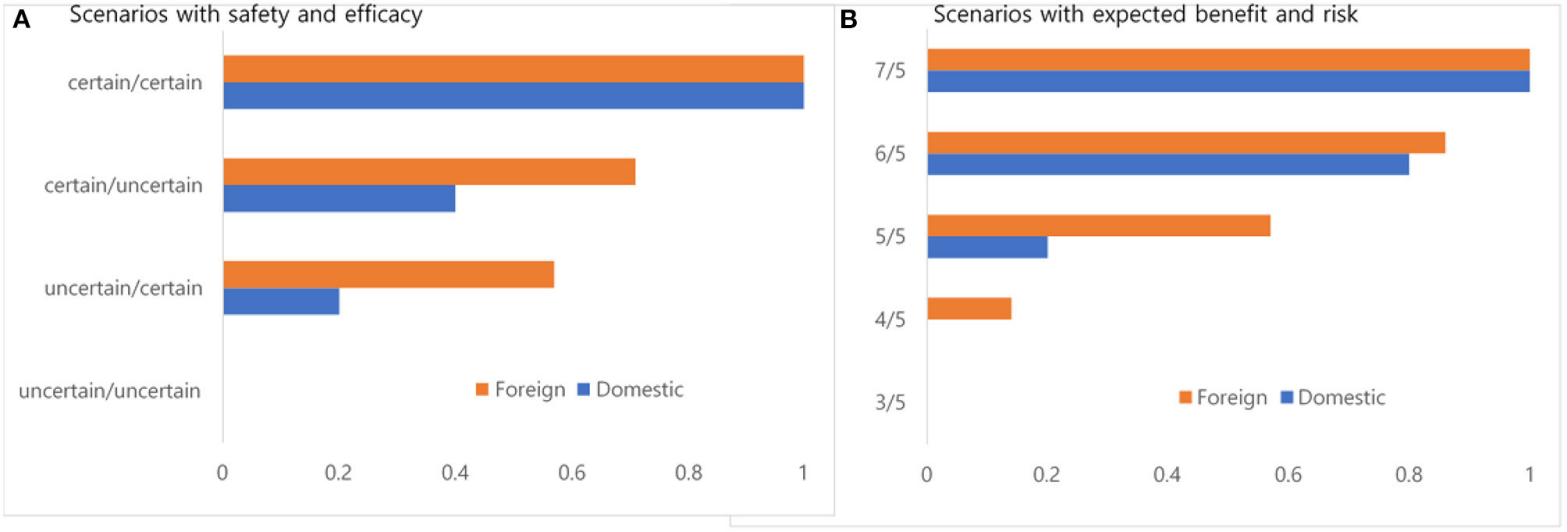
**(A,B)** Degree of consent for new drug approval with two scenarios from the perspectives of safety and efficacy (un)certainty and expected benefits and risks.

Figure 2 describes the degree of consent for new drug approval with two scenarios from the perspectives of uncertainty in safety and efficacy and expected benefits and risks. The manufacturers fully agreed that a new drug with certainty in safety and efficacy would be eligible for market approval. In contrast, they fully disagreed that a new drug with uncertainty in safety and efficacy would be eligible for market approval. Similarly, the manufacturers fully agreed that new drugs in which the expected benefits outweighed the expected risks by two units would be eligible for market approval. In contrast, they fully disagreed that new drugs in which the expected risks outweighed the expected benefits by two units would be eligible for market approval. For each scenario, foreign manufacturers were more likely to accept market approval of a new drug.

Table 3 presents the survey results for the decision-making structure, transparency, regulation, and stability. We separated the decision-making structure into the MFDS and an advisory committee and asked about their expertise and conflicts of interest. The manufacturers disagreed that the MFDS had enough human resources to review new drug applications. However, they agreed that the MFDS had expertise in regulatory decisions. Variations were also noted. The foreign manufacturers' perceptions toward conflicts of interest of the MFDS were negative, while that of domestic manufacturers were positive. Similarly, the foreign manufacturers' perceptions toward expertise and conflicts of interest of an advisory board were negative, while that of the domestic manufacturers were neutral.

**Table 3:** Survey results on decision structure, transparency, regulation, and stability.

**Domains**	**Questions**	**Domestic company *N* = 5**	**Foreign company *N* = 7**
Decision structure	MFDS has enough human resources to review new drug applications	−0.60	−1.43
	MFDS has expertise in regulatory decisions	0.80	1.00
	MFDS is independent of conflicts of interest	1.20	−0.71
	An advisory committee has expertise in regulatory decisions	0.40	−0.43
	An advisory committee is independent of conflicts of interest	0.00	−0.57
Transparency	The authority notices regulatory decisions	1.20	−0.43
	The authority notices the underlying reasons for the regulatory decisions	0.20	−0.71
	The authority explains the regulatory decisions	0.00	−1.00
	The authority explains the underlying reasons for regulatory decisions	−0.20	−0.86
Regulation	The authority effectively manages uncertainty in safety	0.60	−0.43
	The authority effectively manages uncertainty in efficacy	0.20	−0.57
Stability	Laws and regulations on regulatory systems are stable	−0.20	−0.14
	Regulatory decisions are predictable	0.00	−0.57
	Regulatory decisions are consistent with previous decisions	0.60	−0.71

*MFDS, Ministry of Food and Drug Safety*.

## Discussion

Regulatory agencies have evolved to enhance their reputations in decision-making. Meanwhile, manufacturers and patient organizations and have requested open decision-making processes for regulatory decisions to guarantee the timely market approval of new drugs (13–15). In these contexts, this study evaluated the perceptions of regulatory decision-making for new drugs from the viewpoints of the manufacturers in South Korea. Results from this study could provide evidence on establishing politically legitimate regulatory decision-making processes for new drugs approval.

### Regulatory Decisions as a Technical Merit

The manufacturers perceived that a regulatory decision made by the MFDS was solely based on technical merit. They responded that safety, efficacy, and benefit-to-harm ratio were the most relevant criteria and the most prioritized criteria in regulatory decision-making. Furthermore, they indicated that the MFDS was the sole relevant member in a regulatory decision-making.

As already explained, we conducted this study as part of a larger study on the perceptions of manufacturers in the decision-making process for adopting new drugs. The previously published study regarding reimbursement decisions was noteworthy in comparing the perceptions of manufacturers on new drug approval and new drug reimbursement (25). We asked the same survey items regarding the decision-making criteria and the participation of stakeholders in the decision-making process. The employees of manufacturers who had at least 10 years of working experience in health technology assessment emphasized integrated and comprehensive health technology assessments (25). They argued that various criteria, including disease severity, burden of disease, and equity, could be considered in reimbursement decision-making and various stakeholders could voice their opinions or participate under an integrated and comprehensive health technology system. In contrast, employees in manufacturers who had expertise in regulatory affairs perceived that a regulatory decision was made solely by the MFDS within a more closed decision-making system.

Similar to reimbursement decision-making, regulatory decision-making present high uncertainty and high political stakes (6). Manufacturers are required to demonstrate “substantial evidence” regarding the safety and efficacy of new drugs. Substantial evidence means “evidence consisting of adequate and well-controlled investigations, including clinical investigations, by experts qualified by scientific training and experience to evaluate the effectiveness of the drug involved, on the basis of which it could fairly and reasonably be concluded by such experts that the drug will have the effect it purports or is represented to have… (26)”. However, regulatory reviews based on “substantial evidence” could be reversed in certain circumstance (27, 28). Furthermore, the concept of “substantial evidence” through “adequate and well-controlled investigations” have evolved after the implementation of the 21st Century Cures Act in the United States (29, 30). In this circumstance, it is reasonable to assume that regulatory decisions are occasionally determined by the agency given the political benefits and/or costs that organized stakeholders imposed in regulatory decisions (5).

### Regulatory Body and Advisory Committee

In this study, several interesting findings on the regulatory agency and advisory committee were noted. The manufacturers agreed on a lack of human resources in the agency for reviewing new drug applications. For instance, foreign manufacturers were very negative toward the survey item that the MFDS had enough human resources to review applications. Their negative responses shed light on the issue of recruiting additional human resources and/or the retention of human resources within the agency. The manufacturers agreed that the agency had expertise in regulatory decisions despite a lack of human resources. However, the foreign manufacturers disagreed that the agency was independent of conflicts of interest. This finding might seem to be partially associated with political considerations or factors other than scientific evidence in regulatory decisions. As we already explained, the agency cannot make decisions on the sole basis of scientific evidence (31). The regulatory decisions, similar to reimbursement decisions, are made under intertwined contexts, including science, values, and politics (31).

The foreign manufacturers disagreed that the advisory committee had expertise in regulatory decisions and that the committee was independent of conflicts of interest. Conflicts of interest of the advisory committees are not new to the regulatory decision-making process (32, 33). The advisory committee was devised to provide external expertise in regulatory decision-making. However, the manufacturers perceived that the committee did not have enough expertise in regulatory decision-making. The negative perception of foreign manufacturers seemed to be partially associated with the lack of transparency in the regulatory decision-making process. The foreign manufacturers understood that the authority could seek the aid of the advisory committee to supplement its expertise. However, they argued that the underlying reasons for the decisions were not well explained. A few of them indicated that few members of the advisory committee could not understand the submitted evidence from the perspectives of regulatory affairs, clinical background, and statistics.

In addition to an advisory committee, public involvement in terms of deliberative and participatory democracy has been requested to make politically legitimate regulatory decisions (14). An advisory committee in the regulatory agency includes various members from academics, professionals, manufacturers, consumers, and patients. For instance, the Food and Drug Administration (FDA) has made significant efforts to expand the role of patients in regulatory decision-making and responded to the opinions of patients and their caregivers (34). The MFDS has also tried to expand the role of patients in its decision-making (35). Consistent with these efforts, the foreign manufacturers responded that the participation of patients on an advisory board was relevant. The FDA was required to embrace the idea that citizens could contribute to the deliberation process (13, 36). In this context, we asked about the relevance of the participation of the laypersons in a decision-making body and advisory board. However, the manufacturers disagreed on the participation of the laypersons in the decision-making body or advisory body. They responded that the laypersons could not fully understand the submitted data.

### Variations in Risk Aversion Between Foreign and Domestic Manufacturers

Regulatory agencies approve new drugs based on their assessment of the available evidence. We asked the degree of consent for the market approval of new drugs in various scenarios. When we provided scenarios with uncertainty in safety and efficacy, the manufacturers emphasized certainty in safety more than certainty in efficacy when making regulatory decisions. When we provided scenarios with expected benefits and risks, the foreign manufacturers were more likely to agree with the market approval of new drugs than the domestic manufacturers. Interestingly, the proportion of manufacturers who agreed with market approval was lower than our expectations for the scenario with the same expected benefit and risk values. This finding indicates that manufacturers, in particular domestic manufactures, presented higher risk aversion behavior when making regulatory decisions. This conservative perspective of domestic manufacturers in regulatory decision-making was very similar to that of the regulatory agency (9, 37, 38).

### Study Limitations

This study had limitations. First, this study conducted a survey and interviews designed for manufacturers, implying that the findings from this study were solely based on the perceptions from the viewpoints of the manufacturers. Second, this study included a small sample size. Further research with larger sample size is necessary to validate the study findings reported in this study. It is noteworthy that the number of manufacturers, in particular domestic manufacturers, who had introduced new drugs into the market was limited. Finally, we evaluated regulatory decision-making in South Korea. Our findings and implications could not be generalized to other health systems with different contexts.

## Conclusions

The manufacturers perceived that a regulatory decision made by the MFDS was solely based on technical merit within a closed decision-making system. However, the foreign manufacturers disagreed that the regulatory agency and the advisory committee were independent of conflicts of interest, which might imply that regulatory decisions were occasionally determined by the agency given the political benefits and/or costs within a more open system. The role of an advisory committee in terms of deliberation and participatory democracy were requested to make politically legitimate regulatory decisions from the viewpoints of the manufacturers. However, their perceptions toward public involvement in regulatory decision-making is still at the early stage.

## Data Availability Statement

The raw data supporting the conclusions of this article will be made available by the authors, upon reasonable request.

## Ethics Statement

The studies involving human participants were reviewed and approved by Ewha Womans University (IRB No. EWHA-201904-0010-01). The patients/participants provided their written informed consent to participate in this study.

## Author Contributions

K-BS collected the data. K-BS and SP undertook the analysis and prepared the final manuscript. Both authors contributed to the article and approved the submitted version.

## Funding

This work was supported by the Ministry of Education of the Republic of Korea and the National Research Foundation of Korea (NRF-2019S1A5A8032445). The funding source was not involved in study design, the collection, analysis and interpretation of data, in the writing of the report, and in the decision to submit the article for publication.

## Conflict of Interest

The authors declare that the research was conducted in the absence of any commercial or financial relationships that could be construed as a potential conflict of interest.

## Publisher's Note

All claims expressed in this article are solely those of the authors and do not necessarily represent those of their affiliated organizations, or those of the publisher, the editors and the reviewers. Any product that may be evaluated in this article, or claim that may be made by its manufacturer, is not guaranteed or endorsed by the publisher.
