# Supplementary material for: Perceptions of Regulatory Decision-Making for New Drugs From the Viewpoints of the Manufacturers in South Korea
Source: Front Med (Lausanne). 2022 Mar 30;9:869262. doi: 10.3389/fmed.2022.869262 (PMC9006612; doi:10.3389/fmed.2022.869262)
Supplement: Supplementary file 1 [file Data_Sheet_1.PDF]

1. The following questions are about the criteria in regulatory decision-making

a. What do you think should be the relevant criteria for making a regulatory decision for a new drug? Please, measure on a 5-point scale whether the criteria presented below are relevant as a criterion for a regulatory decision.

A criterion		Very irrelevant	Irrelevant	Moderate	Relevant	Very relevant
Drug	Safety	①	②	③	④	⑤
	Efficacy in clinical trials	①	②	③	④	⑤
	Clinical effectiveness in real world	①	②	③	④	⑤
	Benefit-to-harm ratio	①	②	③	④	⑤
	Consistency of evidence	①	②	③	④	⑤
	Price/cost of treatment	①	②	③	④	⑤
	Cost-effectiveness	①	②	③	④	⑤
	Budget impact	①	②	③	④	⑤
Disease	Disease severity	①	②	③	④	⑤
	Health related quality of life	①	②	③	④	⑤
	Alternative treatments	①	②	③	④	⑤
	Burden of disease	①	②	③	④	⑤
	Patient population	①	②	③	④	⑤
Status in other Countries	Marketing approval in other countries	①	②	③	④	⑤
	Reimbursement status in other countries	①	②	③	④	⑤
	Price in other countries	①	②	③	④	⑤

b. What do you think should be the prioritized criteria for making a regulatory decision for a new drug. Please, select and write only the 1st, 2nd, and 3rd priority criterion presented above.

The 1st priority criteria	The 2nd priority criteria	The 3rd priority criteria

2. The following question is about the participation of various stakeholders in the decision-making process

a. Ideally, do you think that it would be relevant for the stakeholders presented below to participate in a decision-making body or advisory board?

		Decision-making body					Advisory board				
		Very irrelevant	Irrelevant	Moderate	Relevant	Very relevant	Very irrelevant	Irrelevant	Moderate	Relevant	Very relevant
Interest groups	Manufacturers	①	②	③	④	⑤	①	②	③	④	⑤
	Consumer	①	②	③	④	⑤	①	②	③	④	⑤
	Patient	①	②	③	④	⑤	①	②	③	④	⑤
	Laypersons	①	②	③	④	⑤	①	②	③	④	⑤
Expert groups	Physicians	①	②	③	④	⑤	①	②	③	④	⑤
	Toxicologist	①	②	③	④	⑤	①	②	③	④	⑤
	Clinical Pharmacy	①	②	③	④	⑤	①	②	③	④	⑤
	Statistics	①	②	③	④	⑤	①	②	③	④	⑤
	Public Health	①	②	③	④	⑤	①	②	③	④	⑤
Government authority	MFDS	①	②	③	④	⑤	①	②	③	④	⑤
	HIRA	①	②	③	④	⑤	①	②	③	④	⑤
	NHIS	①	②	③	④	⑤	①	②	③	④	⑤
	MOH	①	②	③	④	⑤	①	②	③	④	⑤

Note: MFDS, Ministry of Food and Drug Safety; HIRA, Health Insurance Review and Assessment Service; NHIS, National Health Insurance Service; MOH, Ministry of Health and Welfare

b. Do you think the stakeholders presented below is interested and influential in regulatory decision-making for new drug.

		Interest					Influence				
		Never interested	Not interested	Moderate	Interested	Very Interested	Never influential	Not influential	Moderate	Influential	Very Influential
Interest groups	Manufacturers	①	②	③	④	⑤	①	②	③	④	⑤
	Consumer	①	②	③	④	⑤	①	②	③	④	⑤
	Patient	①	②	③	④	⑤	①	②	③	④	⑤
	Laypersons	①	②	③	④	⑤	①	②	③	④	⑤
Expert groups	Physicians	①	②	③	④	⑤	①	②	③	④	⑤
	Toxicologist	①	②	③	④	⑤	①	②	③	④	⑤
	Clinical Pharmacy	①	②	③	④	⑤	①	②	③	④	⑤
	Statistics	①	②	③	④	⑤	①	②	③	④	⑤
	Public Health	①	②	③	④	⑤	①	②	③	④	⑤
Government authority	MFDS	①	②	③	④	⑤	①	②	③	④	⑤
	HIRA	①	②	③	④	⑤	①	②	③	④	⑤
	NHIS	①	②	③	④	⑤	①	②	③	④	⑤
	MOH	①	②	③	④	⑤	①	②	③	④	⑤

Note: MFDS, Ministry of Food and Drug Safety; HIRA, Health Insurance Review and Assessment Service; NHIS, National Health Insurance Service; MOH, Ministry of Health and Welfare

c. Whose role do you think should be expanded in regulatory decision-making? Please, select and write only the 1st, 2nd, and 3rd priority stakeholder presented above.

The 1st priority stakeholder	The 2nd priority stakeholder	The 3rd priority stakeholder

3. **The following question is about the regulatory decision with several scenarios regarding the characteristics of new drugs.**

a. [Safety and Efficacy] The following scenarios are assumption about safety and efficacy data of a new drug submitted by a manufacturer to the regulatory authority. In this scenario, do you think the new drug should be approved by the authority?

Safety	Efficacy	Regulatory decision	
		Do not approve the drug	Do approve the drug
Certain	Certain		
Certain	Uncertain		
Uncertain	Certain		
Uncertain	Uncertain		

b. [Benefits and Risk] The following scenarios are assumptions about the expected benefits and risks of a new drug submitted by manufacturers to the regulatory authority. In this scenario, do you think the new drug should be approved by the authority?

Expected risk caused by uncertainty	Expected benefits through timely access	Regulatory decision	
		Do not approve the drug	Do approve the drug
5	3		
5	4		
5	5		
5	6		
5	7		

4. The following question is about the decision structure, transparency, regulation, and stability of the decision-making process.

a. Do you agree with the following statements?

		Never agree	Not agree	Moderate	Agree	Very agree
Decision structure	MFDS has enough human resources to review new drug applications	①	②	③	④	⑤
	MFDS has expertise in regulatory decisions	①	②	③	④	⑤
	MFDS is independent of conflicts of interest	①	②	③	④	⑤
	An advisory committee has expertise in regulatory decisions	①	②	③	④	⑤
	An advisory committee is independent of conflicts of interest	①	②	③	④	⑤
Transparency	The authority notices regulatory decisions	①	②	③	④	⑤
	The authority notices the underlying reasons for the regulatory decisions	①	②	③	④	⑤
	The authority explains the regulatory decisions	①	②	③	④	⑤
	The authority explains the underlying reasons for regulatory decisions	①	②	③	④	⑤
Regulation	The authority effectively manages uncertainty in safety	①	②	③	④	⑤
	The authority effectively manages uncertainty in efficacy	①	②	③	④	⑤
Stability	Laws and regulations on regulatory systems are stable	①	②	③	④	⑤
	Regulatory decisions are predictable	①	②	③	④	⑤
	Regulatory decisions are consistent with previous decisions	①	②	③	④	⑤
